# Disparities in oral glucocorticoid prescribing among patients with mental disorders: nationwide cohort study

**DOI:** 10.1192/bjo.2026.12027

**Published:** 2026-07-03

**Authors:** Tak Kyu Oh, In-Ae Song

**Affiliations:** Department of Anesthesiology and Pain Medicine, Seoul National University Bundang Hospital, Seongnam, Republic of Korea; Department of Anesthesiology and Pain Medicine, Seoul National University College of Medicine, Seoul, Republic of Korea

**Keywords:** Glucocorticoids, psychiatric disorders, propensity score matching, pharmacoepidemiology, population-based cohort study

## Abstract

**Background:**

Glucocorticoids are widely prescribed for autoimmune and inflammatory conditions, but their long-term use carries serious risks. Patients with psychiatric disorders have a high burden of medical comorbidities, which may be associated with higher odds of receiving sustained glucocorticoid therapy.

**Aims:**

We examined whether psychiatric patients were more likely to receive sustained oral glucocorticoid therapy compared with controls, and assessed variation across psychiatric subgroups.

**Method:**

This retrospective, nationwide cohort study used South Korea’s National Health Insurance Service database. Adults diagnosed with a major psychiatric disorder in 2021 (*n* = 331 020) were compared with a sample without psychiatric disorders (*n* = 668 980). Propensity score matching generated 283 942 participants per group. The outcome was sustained oral glucocorticoid use in 2022, defined as ≥90 cumulative days with continuous therapy ≥90 days and prescription gaps ≤30 days.

**Results:**

Before propensity score matching, glucocorticoid use in 2022 was more frequent in psychiatric patients (13.8%) than controls (10.8%; odds ratio 1.32, 95% CI 1.31–1.34; *P* < 0.001). After matching, the difference persisted (14.7 *v*. 12.5%; odds ratio 1.18, 95% CI 1.16–1.19; *P* < 0.001). Multivariable analyses confirmed higher odds of glucocorticoid use (odds ratio 1.05, 95% CI 1.03–1.06; *P* < 0.001). Increased risk was observed for anxiety disorders (odds ratio 1.06) and obsessive–compulsive disorder (odds ratio 1.07), whereas major depressive disorder showed no significant association.

**Conclusions:**

Psychiatric patients are more likely to receive sustained glucocorticoid therapy, underscoring the need for cautious prescribing and monitoring in this vulnerable population.

Glucocorticoids remain one of the most widely prescribed classes of medications worldwide because of their potent anti-inflammatory and immunosuppressive effects. They are essential in the management of autoimmune diseases, chronic pulmonary disorders and a wide range of other systemic conditions.^1^ However, chronic or sustained use of glucocorticoids is associated with substantial adverse outcomes, including metabolic complications, osteoporosis, cardiovascular disease and psychiatric side-effects such as mood disturbances and psychosis.^2–4^

Patients with psychiatric disorders constitute a particularly vulnerable population with high rates of physical comorbidities and increased healthcare utilisation.^5,6^ Epidemiologic studies consistently showed that individuals with schizophrenia, bipolar disorder, depression or anxiety disorders have elevated risks of cardiovascular disease, diabetes and chronic pain syndromes compared with the general population.^7,8^ These comorbidities often associated with the need for long-term pharmacologic treatment, raising the likelihood of glucocorticoid exposure. At the same time, glucocorticoids themselves can induce or exacerbate psychiatric symptoms, including depression, mania and psychosis, creating a complex bidirectional relationship between glucocorticoid therapy and mental health.^9,10^

Despite these concerns, limited evidence exists on whether patients with psychiatric disorders are more likely to receive glucocorticoids compared with individuals without such diagnoses. Previous investigations have largely focused on the neuropsychiatric consequences of glucocorticoid therapy, often in selected clinical populations, but have not systematically addressed prescribing patterns across psychiatric subgroups.^2,11,12^ Large-scale, population-based analyses directly comparing glucocorticoid use between psychiatric and non-psychiatric populations are lacking.

To address this gap, we conducted a nationwide retrospective cohort study using the South Korean National Health Insurance Service (NHIS) database. To ensure a clear temporal relationship between psychiatric diagnosis and subsequent medication patterns, we identified patients diagnosed with psychiatric disorders in 2021 and followed their glucocorticoid prescription patterns throughout 2022. Our objective was to examine the comparative prevalence of sustained glucocorticoid use among patients with major psychiatric disorders versus those without, while rigorously controlling for clinical and behavioural factors, including underlying autoimmune conditions, chronic pain, metabolic status and healthcare utilisation frequency. By clarifying these patterns, our findings may provide critical insight for clinicians managing psychiatric populations at heightened risk for glucocorticoid-related complications.

## Method

### Study design and ethical approval

We conducted a retrospective cohort study utilising nationwide administrative health data, and reported it in line with the Strengthening the Reporting of Observational Studies in Epidemiology (STROBE) statement.^13^ Ethical approval was granted by the Institutional Review Board of Seoul National University Bundang Hospital (X-2309-852-901). The NHIS reviewed the study protocol (NHIS-2025-04-1-102) and permitted data access. Since the data-set was anonymised and originally collected for reimbursement and administrative use, the requirement for informed consent from individuals was waived, consistent with Korean regulations and the principles of the Declaration of Helsinki (1975, revised 2008).

### Data source

The analysis relied on the NHIS database, which is South Korea’s nationwide compulsory health insurance programme covering nearly the entire population. This resource includes longitudinal, person-level information on demographics, diagnostic codes, healthcare utilisation, prescriptions, and mortality. Diagnoses are coded according to the ICD-10. Although most clinical care is delivered in private medical settings, mandatory claims reporting to the NHIS and centralised data management ensure high completeness and reliability.^14^

### Study population

Within the NHIS database, all physicians – including psychiatrists – are required to record ICD-10 diagnostic codes for reimbursement, which supports the accuracy of psychiatric diagnoses. We included adults aged ≥18 years who received at least one diagnosis of a major psychiatric disorder in 2021 (exposure period). The psychiatric conditions of interest were schizophrenia spectrum disorders (F20, F23, F23.2, F25), bipolar disorder (F30, F31), autism spectrum disorders (F84.0, F84.1, F84.5, F84.9), eating disorders (F50), alcohol use disorders (F10), attention-deficit hyperactivity disorder (F90), obsessive–compulsive disorder (F42), major depressive disorder (single and recurrent episodes; F32, F33), tic disorders (F95), post-traumatic stress disorder (F43.1) and anxiety disorders (F40, F41). A total of 331 020 individuals satisfied these inclusion criteria (psychiatric disorder group).

**Fig. 1:**
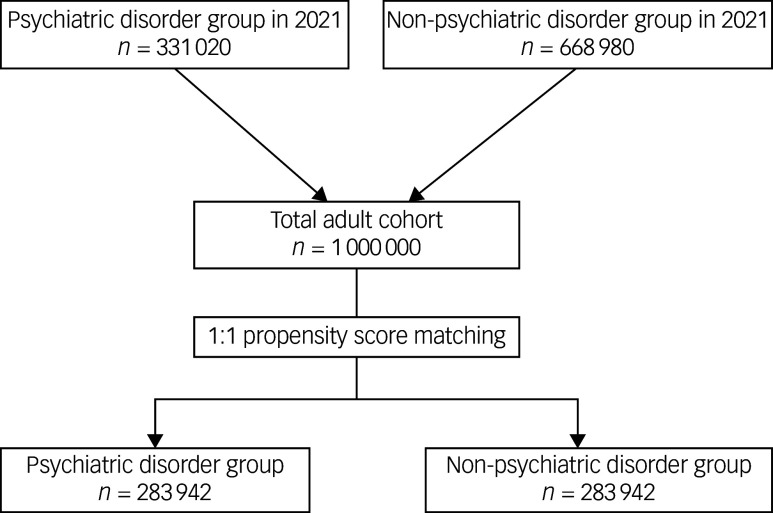
Fig. 1 long description. Flowchart of study population selection.

For the reference cohort, we identified 668 980 adults without any psychiatric diagnoses in 2021 (non-psychiatric disorder group). To ensure a rigorous comparison and minimise confounding, we performed 1:1 propensity score matching between both groups, based on all baseline covariates measured in 2021. The final matched cohort for the primary analysis consisted of 567 884 participants, with 283 942 in each group. Figure 1 illustrates the detailed flow of cohort construction.

### Study outcomes

The primary end-point was to evaluate and compare the sustained use of oral glucocorticoids between the psychiatric disorder and non-psychiatric disorder cohorts during the follow-up period (1 January to 31 December 2022). To ensure a clear temporal sequence, only glucocorticoid prescriptions occurring after the established psychiatric diagnosis in 2021 were considered for the primary outcome analysis.

Sustained use was operationally defined as a cumulative supply of systemic glucocorticoids lasting at least 90 days within the follow-up year. Consistent with previous epidemiological studies, this was further refined as continuous treatment maintained for 90 or more days, permitting prescription gaps of up to 30 days to account for potential variations in medication adherence or refill timing.^1,15^ For participants whose initial glucocorticoid prescription began in the latter part of the follow-up period (after 1 September 2022), prescription records were tracked through 31 March 2023, to ensure a sufficient window for the appropriate classification of sustained exposure.

### Covariates

Baseline characteristics included demographic and socioeconomic indicators. We accounted for age and gender, as well as employment status, residential setting and household income at the index date. In the NHIS system, the lowest-income beneficiaries exempt from insurance premiums are covered by the Medical Aid programme, and the remaining population is distributed into quartiles based on income (quartile 1 being the lowest and quartile 4 being the highest). Residential area was classified as metropolitan (Seoul and other large cities) versus non-metropolitan regions.

Overall morbidity burden was assessed using the Charlson Comorbidity Index (CCI), derived from ICD-10 claims data (Supplementary Table 1). Disability status was also incorporated. In Korea, disability registration requires physician certification and formal review, with severity ratings reflecting functional limitations in daily life. For this analysis, disability was grouped into mild-to-moderate or severe, with detailed categories presented in Supplementary Table 2.

We additionally adjusted for baseline musculoskeletal disorders, autoimmune conditions, and clinical indications and metabolic status that may affect glucocorticoid prescribing, including fibromyalgia, chronic low back pain, other chronic spinal pain, degenerative osteoarthritis, rheumatoid arthritis, headache, trigeminal neuralgia and myofascial pain. Autoimmune conditions were also considered, specifically ankylosing spondylitis, autoimmune hepatitis, Behçet’s disease, Crohn’s disease, polymyositis, polyarteritis, Sjögren syndrome, systemic lupus erythematosus, systemic sclerosis and ulcerative colitis. Moreover, clinical indications and metabolic status included chronic respiratory diseases (asthma and chronic obstructive pulmonary disease (COPD)) and metabolic factors (obesity, dyslipidaemia, and other metabolic disorders) (ICD-10 codes listed in Supplementary Table 3).

### Statistical analysis

Continuous variables were expressed as means with standard deviations, whereas categorical variables were summarised as frequencies with percentages. Although the non-psychiatric disorder cohort was initially generated using age- and gender-based stratification, residual differences in other baseline variables remained. To mitigate these imbalances, additional 1:1 propensity score matching was applied.^16^ Propensity score values were estimated through logistic regression including all predefined covariates, and nearest-neighbour matching without replacement was used, with a caliper width set to 0.25 times the standard deviation of the logit of the propensity score. Covariate balance before and after matching was assessed using absolute standardised mean differences, with thresholds <0.1 considered acceptable.

Primary outcomes (sustained systemic glucocorticoid use) were evaluated using conditional logistic regression models for the matched cohort to account for the paired nature of the data. Results are presented as odds ratios with 95% CIs. We also fitted multivariable logistic regression models in the total population, adjusting for all covariates and incorporating specific psychiatric diagnoses – including major depressive disorder (single and recurrent episodes), anxiety disorder, post-traumatic stress disorder and eating disorders – as separate predictors. Multicollinearity was assessed using variance inflation factors (<2.0), and model calibration was verified using the Hosmer–Lemeshow test.

To evaluate the relationship between physical comorbidities and healthcare-seeking behaviour, we examined the correlation between the CCI and the total number of out-patient clinic visits by using Spearman’s rank correlation analysis. The analysis revealed a moderate correlation (rho = 0.48, *P* < 0.001), confirming that these variables capture distinct clinical constructs. Furthermore, to avoid potential overadjustment bias, our primary propensity score model excluded out-patient visit frequency, as healthcare utilisation was identified as a plausible mediator rather than a baseline confounder on the causal pathway between psychiatric status and subsequent prescribing. A sensitivity analysis including visit frequency was conducted to assess the robustness of the findings (Supplementary Tables 4 and 5). Additionally, *E*-values were calculated to quantify the strength of unmeasured confounding required to nullify our observed associations.

Subgroup analyses were conducted to evaluate the robustness of the findings according to gender, age (<65 *v*. ≥65 years), and the presence of underlying musculoskeletal diseases, autoimmune conditions or chronic respiratory diseases (COPD or asthma). All statistical analyses were carried out using R software version 4.5.1 for Windows (R Foundation for Statistical Computing, Vienna, Austria; see https://www.r-project.org/foundation/), and two-sided *P*-values < 0.05 were regarded as statistically significant.

## Results

### Study population

Table 1 summarises the baseline demographic and clinical features of participants in the psychiatric disorder and non-psychiatric disorder groups, both before and following propensity score matching. After matching, all covariates showed absolute standardised mean differences below 0.1, indicating adequate balance between the two cohorts.

**Table 1:** Baseline demographic and clinical features of participants in the psychiatric disorder and non-psychiatric disorder groups, before and after propensity score matching Table 1 long description.

Variable	Before propensity score matching		ASD	After propensity score matching		ASD
	Psychiatric disorder group (*n* = 331 020)	Non-psychiatric disorder group (*n* = 668 980)		Psychiatric disorder group (*n* = 283 942)	Non-psychiatric disorder group (*n* = 283 942)	
	*n* (%)^a^	*n* (%)		*n* (%)	*n* (%)	
Age, years, mean (s.d.)	60.8 (18.9)	50.6 (17.9)	0.554	59.0 (19.0)	58.7 (17.8)	0.015
Gender, male	159 105 (48.1)	340 895 (51.0)	0.058	137 193 (48.3)	137 532 (48.4)	0.002
Residence			0.019			0.009
Urban area	139 397 (42.1)	288 048 (43.1)		120 909 (42.6)	122 232 (43.0)	
Rural area	191 623 (57.9)	380 932 (56.9)		163 033 (57.4)	161 710 (57.0)	
Household income level			0.391			0.091
Medical aid programme	41 777 (12.6)	18 222 (2.7)		24 159 (8.5)	17 604 (6.2)	
Quartile 1 (lowest)	64 800 (19.6)	138 364 (20.7)		58 076 (20.5)	60 534 (21.3)	
Quartile 2	52 833 (16.0)	130 831 (19.6)		48 285 (17.0)	49 050 (17.3)	
Quartile 3	63 722 (19.3)	159 608 (23.9)		58 134 (20.5)	58 335 (20.5)	
Quartile 4 (highest)	102 907 (31.1)	208 409 (31.2)		90 731 (32.0)	94 203 (33.2)	
Unknown	4981 (1.5)	13 546 (2.0)		4557 (1.6)	4216 (1.5)	
Having a job	188 222 (56.9)	449 213 (67.1)	0.213	170 513 (60.1)	174 241 (61.4)	0.027
Underlying disability			0.391			0.071
Mild to moderate	30 545 (9.2)	26 137 (3.9)		22 177 (7.8)	20 796 (7.3)	
Severe	28 478 (8.6)	12 257 (1.8)		16 416 (5.8)	12 220 (4.3)	
CCI, point, mean (s.d.)	3.6 (3.1)	1.9 (2.4)	0.621	3.3 (2.9)	3.2 (2.8)	0.029
Myocardial infarction	15 083 (4.6)	13 565 (2.0)	0.142	11 104 (3.9)	10 365 (3.7)	0.014
Congestive heart failure	59 254 (17.9)	48 574 (7.3)	0.325	42 786 (15.1)	39 682 (14.0)	0.031
Peripheral vascular disease	94 724 (28.6)	93 033 (13.9)	0.365	72 705 (25.6)	71 623 (25.2)	0.009
Cerebrovascular disease	84 174 (25.4)	60 944 (9.1)	0.442	59 260 (20.9)	53 952 (19.0)	0.047
Dementia	70 291 (21.2)	33 363 (5.0)	0.496	43 792 (15.4)	33 231 (11.7)	0.091
Chronic pulmonary disease	138 721 (41.9)	187 141 (28.0)	0.294	110 924 (39.1)	110 053 (38.8)	0.006
Rheumatic disease	36 529 (11.0)	41 716 (6.2)	0.171	28 673 (10.1)	28 287 (10.0)	0.005
Peptic ulcer disease	120 023 (36.3)	154 503 (23.1)	0.291	97 061 (34.2)	99 040 (34.9)	0.015
Mild liver disease	155 923 (47.1)	197 606 (29.5)	0.367	126 208 (44.4)	130 123 (45.8)	0.028
Diabetes without chronic complication	144 752 (43.7)	162 503 (24.3)	0.419	114 144 (40.2)	115 027 (40.5)	0.006
Diabetes with chronic complication	42 237 (12.8)	38 166 (5.7)	0.246	31 637 (11.1)	29 900 (10.5)	0.020
Hemiplegia or paraplegia	10 247 (3.1)	6403 (1.0)	0.152	6810 (2.4)	5575 (2.0)	0.030
Renal disease	18 725 (5.7)	16 327 (2.4)	0.164	13 699 (4.8)	12 439 (4.4)	0.021
Cancer	50 431 (15.2)	59 928 (9.0)	0.193	40 018 (14.1)	39 813 (14.0)	0.002
Moderate or severe liver disease	2445 (0.7)	2136 (0.3)	0.058	1813 (0.6)	1699 (0.6)	0.005
Metastatic solid tumour	6027 (1.8)	7556 (1.1)	0.057	4922 (1.7)	4910 (1.7)	<0.001
HIV/AIDS	511 (0.2)	567 (0.1)	0.020	387 (0.1)	375 (0.1)	0.001
Underlying MSD						
Fibromyalgia	11 173 (3.4)	9878 (1.5)	0.124	8135 (2.9)	7659 (2.7)	0.010
Chronic low back pain	108 010 (32.6)	148 453 (22.2)	0.236	87 679 (30.9)	88 491 (31.2)	0.006
Other chronic spine pain	62 020 (18.7)	84 012 (12.6)	0.171	50 728 (17.9)	51 164 (18.0)	0.004
Degenerative osteoarthritis	123 243 (37.2)	148 861 (22.3)	0.332	98 422 (34.7)	98 849 (34.8)	0.003
Rheumatoid arthritis	21 730 (6.6)	26 253 (3.9)	0.119	17 259 (6.1)	16 971 (6.0)	0.004
Headache	53 819 (16.3)	54 081 (8.1)	0.252	41 613 (14.7)	41 508 (14.6)	0.001
Trigeminalgia	4834 (1.5)	4287 (0.6)	0.080	3590 (1.3)	3302 (1.2)	0.009
Myofascial pain	118 029 (35.7)	187 197 (28.0)	0.165	98 520 (34.7)	100 332 (35.3)	0.013
Other analgesics use						
Paracetamol	115 615 (34.9)	146 686 (21.9)	0.291	92 632 (32.6)	92 833 (32.7)	0.002
NSAIDs	24 745 (7.5)	35 121 (5.2)	0.091	20 170 (7.1)	20 180 (7.1)	<0.001
Other potential glucocorticoid indication						
Ankylosing spondylitis	3185 (1.0)	3988 (0.6)	0.042	2599 (0.9)	2627 (0.9)	0.001
Autoimmune hepatitis	1985 (0.6)	2415 (0.4)	0.035	1604 (0.6)	1664 (0.6)	0.003
Bechet’s disease	602 (0.2)	624 (0.1)	0.024	464 (0.2)	480 (0.2)	0.001
Crohn’s disease	718 (0.2)	958 (0.1)	0.017	575 (0.2)	593 (0.2)	0.001
Polymyositis	394 (0.1)	395 (0.1)	0.020	285 (0.1)	268 (0.1)	0.002
Polyarteritis	427 (0.1)	455 (0.1)	0.019	338 (0.1)	322 (0.1)	0.002
Shogren syndrome	2372 (0.7)	1813 (0.3)	0.064	1675 (0.6)	1482 (0.5)	0.009
Systemic lupus erythematosus	3133 (0.9)	3703 (0.6)	0.046	2485 (0.9)	2462 (0.9)	0.001
Systemic sclerosis	203 (0.1)	237 (0.0)	0.012	167 (0.1)	163 (0.1)	0.001
Ulcerative colitis	1288 (0.4)	1694 (0.3)	0.024	1067 (0.4)	1034 (0.4)	0.002
Obesity	1040 (0.3)	961 (0.1)	0.036	843 (0.3)	827 (0.3)	0.001
Dyslipidaemia	194 319 (58.7)	222 543 (33.3)	0.528	155 945 (54.9)	160 891 (56.7)	0.035
Other metabolic disorders	1540 (0.5)	1214 (0.2)	0.050	1089 (0.4)	1011 (0.4)	0.005
Asthma	36 200 (10.9)	34 355 (5.1)	0.215	26 774 (9.4)	24 979 (8.8)	0.022
COPD	11 079 (3.3)	7397 (1.1)	0.152	7639 (2.7)	6492 (2.3)	0.026

ASD, absolute standardised mean difference; CCI, Charlson Comorbidity Index; MSD, musculoskeletal disease; NSAIDs, non-steroidal anti-inflammatory drugs; COPD, chronic obstructive pulmonary disease.

a. Data are presented as *n* (%) unless stated otherwise.

### Analyses in the propensity score-matched cohort

As shown in Table 2, before propensity score matching, the prevalence of sustained systemic glucocorticoid use in 2022 was 13.8% in the psychiatric disorder group compared with 10.8% in the non-psychiatric disorder group, corresponding to an odds ratio of 1.32 (95% CI 1.31–1.34; *P* < 0.001). After matching, the proportion of sustained glucocorticoid users remained significantly higher among patients with psychiatric disorders (14.7%) than among controls (12.5%), with an odds ratio of 1.18 (95% CI 1.16–1.19; *P* < 0.001). To quantify the potential impact of unmeasured confounding, the *E*-value for this primary association was calculated as 1.64 for the point estimate and 1.59 for the lower bound of the 95% CI. In a sensitivity analysis that included out-patient visit frequency in the propensity score matching model (Supplementary Table 4), the association remained significant, although slightly attenuated (odds ratio 1.13, 95% CI 1.11–1.15; *P* < 0.001; Supplementary Table 5).

**Table 2:** Analyses before and after propensity score matching Table 2 long description.

Outcome	Event (%)	Odds ratio (95% CI)	*P*-value
Before propensity score matching			
Glucocorticoid use			
Non-psychiatric disorder group	72 130/668 980 (10.8)	1	
Psychiatric disorder group	45 664/331 020 (13.8)	1.32 (1.31–1.34)	<0.001
After propensity score matching			
Glucocorticoid use			
Non-psychiatric disorder group	35 492/283 942 (12.5)	1	
Psychiatric disorder group	41 739/283 942 (14.7)	1.18 (1.16–1.19)	<0.001

### Analyses in entire cohort

Results from the multivariable logistic regression are presented in Table 3. In the fully adjusted model (model 1), psychiatric disorders were independently associated with higher glucocorticoid use (odds ratio 1.05, 95% CI 1.03–1.06; *P* < 0.001). Detailed odds ratios for all other covariates in model 1 are provided in Supplementary Table 6. Strong independent risk factors for sustained glucocorticoid use included rheumatoid arthritis (odds ratio 1.77), COPD (odds ratio 1.73), asthma (odds ratio 1.42) and obesity (odds ratio 1.31). Detailed odds ratios for all other covariates in model 1 are provided in Supplementary Table 6.

**Table 3:** Multivariable logistic regression analyses examining associations between specific psychiatric disorders and each outcome Table 3 long description.

Outcome	Odds ratio (95% CI)	*P*-value
Psychiatric disorder group (versus non-psychiatric disorder group) (model 1)	1.05 (1.03–1.06)	<0.001
Psychiatric disease in detail (model 2)		
ADHD	0.99 (0.92–1.06)	0.753
Alcohol use disorder	0.90 (0.85–0.95)	<0.001
Eating disorder	0.98 (0.81–1.18)	0.831
Anxiety disorder	1.06 (1.04–1.08)	<0.001
Autism	0.94 (0.76–1.15)	0.526
Bipolar disorder	0.92 (0.89–0.95)	<0.001
Major depressive disorder (single episode)	1.00 (0.99–1.02)	0.605
Major depressive disorder (recurrent episode)	1.03 (0.98–1.08)	0.245
OCD	1.07 (1.00–1.15)	0.066
Schizophrenia	0.80 (0.77–0.84)	<0.001
Tic	0.99 (0.87–1.12)	0.847
PTSD	1.07 (0.93–1.23)	0.368

ADHD, attention-deficit hyperactivity disorder; OCD, obsessive–compulsive disorder; PTSD, post-traumatic stress disorder.

When examined by individual diagnosis (model 2), significantly elevated odds of sustained glucocorticoid exposure were observed in anxiety disorders (odds ratio 1.06, 95% CI 1.04–1.08; *P* < 0.001) and obsessive–compulsive disorder (odds ratio 1.07, 95% CI 1.00–1.15; *P* = 0.066). By contrast, lower glucocorticoid use was observed in schizophrenia (odds ratio 0.80; *P* < 0.001), bipolar disorder (odds ratio 0.92; *P* < 0.001) and alcohol use disorder (odds ratio 0.90; *P* < 0.001).

### Subgroup analyses

Subgroup analyses (Table 4) revealed consistent associations across genders, with both men (odds ratio 1.03, 95% CI 1.01–1.05; *P* = 0.002) and women (odds ratio 1.04, 95% CI 1.02–1.06; *P* < 0.001) showing significantly higher odds of glucocorticoid use. The association was significant among individuals aged <65 years (odds ratio 1.07, 95% CI 1.05–1.09; *P* < 0.001), but not in those 65 years or older (odds ratio 1.01, 95% CI 0.99–1.03; *P* = 0.333).

**Table 4:** Subgroup analyses Table 4 long description.

Outcome	Odds ratio (95% CI)	*P*-value
Male gender (*n* = 500 000)		
Non-psychiatric disorder group	1	
Psychiatric disorder group	1.03 (1.01–1.05)	0.002
Female gender (*n* = 500 000)		
Non-psychiatric disorder group	1	
Psychiatric disorder group	1.04 (1.02–1.06)	<0.001
Age ≥65 years (*n* = 313 568)		
Non-psychiatric disorder group	1	
Psychiatric disorder group	1.01 (0.99–1.03)	0.333
Age <65 years (*n* = 686 432)		
Non-psychiatric disorder group	1	
Psychiatric disorder group	1.07 (1.05–1.09)	<0.001
Underlying autoimmune disease (*n* = 26 559)		
Non-psychiatric disorder group	1	
Psychiatric disorder group	0.95 (0.90–1.01)	0.124
No underlying autoimmune disease (*n* = 973 441)		
Non-psychiatric disorder group	1	
Psychiatric disorder group	1.04 (1.02–1.05)	<0.001
Underlying chronic pain (*n* = 587 896)		
Non-psychiatric disorder group	1	
Psychiatric disorder group	1.04 (1.02–1.05)	<0.001
No underlying chronic pain (*n* = 412 104)		
Non-psychiatric disorder group	1	
Psychiatric disorder group	1.07 (1.03–1.10)	<0.001
Underlying COPD or asthma (*n* = 78 600)		
Non-psychiatric disorder group	1	
Psychiatric disorder group	0.99 (0.95–1.03)	0.512
No underlying COPD or asthma (*n* = 921 400)		
Non-psychiatric disorder group	1	
Psychiatric disorder group	1.04 (1.03–1.06)	<0.001

COPD, chronic obstructive pulmonary disease.

Psychiatric patients without autoimmune diseases (odds ratio 1.04, 95% CI 1.02–1.05; *P* < 0.001) and those with chronic pain (odds ratio 1.04, 95% CI 1.02–1.06; *P* < 0.001) also showed significantly higher odds of sustained glucocorticoid use.

## Discussion

In this large-scale nationwide cohort study, we found that patients with psychiatric disorders were significantly more likely to use sustained systemic glucocorticoids than those without psychiatric diagnoses. After rigorous propensity score matching, the psychiatric disorder group still showed an 18% higher likelihood of sustained glucocorticoid use. Crucially, by identifying psychiatric diagnoses in 2021 and tracking glucocorticoid use throughout 2022, we established a clear temporal sequence that suggests psychiatric status serves as an independent factor for subsequent long-term steroid prescribing. Although residual confounding cannot be entirely ruled out, this association remained significant after adjusting for measured demographic and clinical factors, and its robustness was further supported by *E*-value analysis. Subgroup analyses further revealed that gender, age and underlying physical conditions modified these associations, with the strongest relative effect observed among patients aged <65 years and those without autoimmune diseases.

Our findings expand upon prior work that has predominantly examined the neuropsychiatric consequences of glucocorticoid treatment rather than differential prescribing patterns. Previous research has consistently shown that glucocorticoids can induce psychiatric symptoms, ranging from mood disturbances and anxiety to frank psychosis.^4,11,12^ A recent meta-analysis further quantified these risks, reporting significant increases in affective, cognitive and behavioural complications associated with glucocorticoid exposure.^17^ Mechanistic reviews suggest that dysregulation of the hypothalamic-pituitary-adrenal axis and altered neurotransmitter signalling may underlie these effects.^18^

In terms of prescribing prevalence, national registry-based studies have shown wide variation across countries. In France, the prevalence of oral glucocorticoid prescriptions in adults approached 17% annually,^1^ whereas in a 17-year cohort analysis the mean annual prevalence remained approximately 3.8%.^19^ A large USA study similarly found that 21% of adults received oral corticosteroids during a 3-year period, with even short-term use associated with increased risks of sepsis, venous thromboembolism and fracture.^20^ Our data add to this literature by showing that individuals with psychiatric diagnoses, a clinically vulnerable group, have significantly higher odds of receiving sustained glucocorticoid therapy compared with those without psychiatric diagnoses.

Several explanations may account for the observed association. First, psychiatric patients have elevated burdens of chronic physical illness – including cardiovascular disease, diabetes and musculoskeletal disorders – that are frequently managed with anti-inflammatory therapy.^7,8^ Furthermore, our multivariable model incorporated several clinical and metabolic factors, such as rheumatoid arthritis (odds ratio 1.77), COPD (odds ratio 1.73), asthma (odds ratio 1.42) and obesity (odds ratio 1.31), which were identified as strong independent risk factors for glucocorticoid use. The fact that the association between psychiatric disorders and glucocorticoid use persisted even after adjusting for these potent clinical indications – with an *E*-value of 1.64 – reinforces the robustness of our results against unmeasured confounding.

Second, the role of healthcare utilisation in this association warrants careful interpretation. Although increased clinic visits provide more opportunities for prescribing, our analysis suggests that visit frequency acts as a mediator rather than a simple baseline confounder. This is evidenced by the increase in the odds ratio from 1.13 (in the model including visit frequency; Supplementary Table 5) to 1.18 when healthcare utilisation was treated as a mediator. Furthermore, the moderate Spearman’s correlation (rho = 0.48) between the CCI and visit frequency confirms that these variables capture distinct clinical constructs.

Third, provider-level variation in prescribing practices has been documented, with some clinicians more likely to continue glucocorticoid therapy over time.^21^ Finally, there may be shared biological pathways linking psychiatric disorders and immune dysregulation. Our findings align with the principles of immunopsychiatry, suggesting that conditions like major depression and anxiety are characterised by chronic activation of the hypothalamic-pituitary-adrenal axis and elevated levels of pro-inflammatory cytokines.^18^ These mechanisms not only increase the risk of inflammatory physical diseases, but also create a common underlying pathophysiology that may be associated with the clinical need for subsequent glucocorticoid treatment.

Interestingly, we found marked heterogeneity across psychiatric diagnoses. Anxiety disorders (odds ratio 1.06) and obsessive–compulsive disorder (odds ratio 1.07) were associated with elevated glucocorticoid use. By contrast, schizophrenia (odds ratio 0.80), bipolar disorder (odds ratio 0.92) and alcohol use disorder (odds ratio 0.90) showed lower prescribing rates. These differences may reflect disparities in healthcare access and the phenomenon of ‘diagnostic overshadowing’, where physical complaints in patients with severe mental illness are frequently overlooked or attributed to their psychiatric condition.^6,22,23^ Conversely, anxiety and depression are associated with higher somatic symptom reporting and treatment-seeking behaviours, potentially contributing to increased glucocorticoid prescribing.

Our findings highlight the need for clinicians to be vigilant when prescribing glucocorticoids to psychiatric patients. Given the well-documented risk of steroid-induced mood and psychotic symptoms,^9,11,12^ prescribers should carefully balance risks and benefits, consider steroid-sparing alternatives and implement proactive psychiatric monitoring during therapy. From a policy perspective, these results emphasise the importance of integrating mental health considerations into routine care for chronic inflammatory conditions, ensuring that psychiatric patients do not experience either overtreatment or undertreatment with glucocorticoids.

Several limitations should be noted. First, although the use of a large, nationwide administrative database provided statistical power and representativeness, reliance on claims data may have introduced misclassification of diagnoses and prescription records. Specifically, certain variables like obesity may be underrecorded in reimbursement databases compared with clinical records. Second, although we established a temporal relationship between 2021 and 2022 and utilised *E*-values to quantify the potential impact of unmeasured factors, residual confounding – such as illness severity, prescriber practice patterns or patient adherence – cannot be entirely excluded. Third, our data did not include information on the precise dosage, cumulative exposure levels or specific clinical indications for each glucocorticoid prescription, which may have influenced both prescribing patterns and clinical outcomes. Fourth, our measure of healthcare utilisation was primarily focused on the frequency of out-patient clinic visits. This metric may not fully capture the complexity of healthcare-seeking behaviours, such as emergency department visits, in-patient admissions or consultations across multiple specialties, which could also play a role in glucocorticoid prescribing patterns. Finally, as an observational study, causal inference cannot be established, and the observed associations should be interpreted as hypothesis-generating, warranting further prospective or mechanistic investigations.

In conclusion, in this nationwide cohort analysis, patients with psychiatric disorders had a higher likelihood of sustained oral glucocorticoid use compared with individuals without such diagnoses, although associations differed across specific psychiatric conditions. These findings underscore the need for heightened clinical vigilance when managing psychiatric populations, who may face both increased prescribing exposure and vulnerability to adverse effects. Future investigations should clarify the mechanisms driving these prescribing patterns and assess targeted interventions to promote safer and more equitable glucocorticoid use in patients with mental illness.

## Supporting information

10.1192/bjo.2026.12027.sm001 Oh and Song supplementary material 1 Oh and Song supplementary material

10.1192/bjo.2026.12027.sm002 Oh and Song supplementary material 2 Oh and Song supplementary material

10.1192/bjo.2026.12027.sm003 Oh and Song supplementary material 3 Oh and Song supplementary material

10.1192/bjo.2026.12027.sm004 Oh and Song supplementary material 4 Oh and Song supplementary material

10.1192/bjo.2026.12027.sm005 Oh and Song supplementary material 5 Oh and Song supplementary material

10.1192/bjo.2026.12027.sm006 Oh and Song supplementary material 6 Oh and Song supplementary material

## Data Availability

The data that support the findings of this study are available from the corresponding author, I.-A.S., upon reasonable request.

## Supplementary material

The supplementary material is available online at https://doi.org/10.1192/bjo.2026.12027

## Fig. 1 Long description

The flowchart begins with two groups: the Psychiatric disorder group with 331,020 individuals and the Non-Psychiatric disorder group with 668,980 individuals. These groups converge into a Total adult cohort of 1,000,000 individuals. The next step involves 1:1 propensity score matching, resulting in two matched groups: the psychiatric disorder group with 283,942 individuals and the Non-psychiatric disorder group with 283,942 individuals.

Navigate back to Fig. 1.

## Table 1 Long description

The table presents a comparison of baseline demographic and clinical features between psychiatric disorder and non-psychiatric disorder groups, both before and after propensity score matching. It includes variables such as age, gender, residence, household income level, employment status, underlying disabilities, and various health conditions. The table is divided into two main sections: before and after propensity score matching, each with columns for the psychiatric disorder group, non-psychiatric disorder group, and absolute standardised mean differences (ASD). Notable trends include differences in age, gender distribution, residence, and various health conditions between the two groups before matching, which are balanced out after matching. All covariates show absolute standardised mean differences below 0.1 after matching, indicating adequate balance between the two cohorts.

Navigate back to Table 1.

## Table 2 Long description

The table presents data on the prevalence of sustained systemic glucocorticoid use in 2022, comparing psychiatric and non-psychiatric disorder groups before and after propensity score matching. It includes four rows and four columns. The columns are labeled Outcome, Event (percentage), Odds ratio (95% CI), and P-value. The rows are divided into two main sections: before propensity score matching and after propensity score matching. Each section contains data for the non-psychiatric disorder group and the psychiatric disorder group. Before matching, the prevalence of glucocorticoid use was 10.8% in the non-psychiatric disorder group and 13.8% in the psychiatric disorder group, with an odds ratio of 1.32 and a P-value of less than 0.001. After matching, the prevalence was 12.5% in the non-psychiatric disorder group and 14.7% in the psychiatric disorder group, with an odds ratio of 1.18 and a P-value of less than 0.001. The table highlights a significantly higher prevalence of glucocorticoid use in the psychiatric disorder group both before and after matching.

Navigate back to Table 2.

## Table 3 Long description

A table with nine rows and three columns presents the results of multivariable logistic regression analyses examining associations between specific psychiatric disorders and glucocorticoid use. The columns are labeled Outcome, Odds ratio (95% CI), and P-value. The first row compares the psychiatric disorder group to the non-psychiatric disorder group, showing an odds ratio of 1.05 with a P-value of less than 0.001. Subsequent rows detail specific psychiatric diseases, including ADHD, alcohol use disorder, eating disorder, anxiety disorder, autism, bipolar disorder, major depressive disorder (single and recurrent episodes), OCD, schizophrenia, tic, and PTSD. Each row lists the odds ratio with a 95% confidence interval and the corresponding P-value, highlighting significant associations where applicable.

Navigate back to Table 3.

## Table 4 Long description

The table presents subgroup analyses of glucocorticoid use, comparing various demographics and health conditions. It includes data for male and female genders, age groups, presence of underlying autoimmune diseases, chronic pain, and chronic obstructive pulmonary disease or asthma. The table has six rows and three columns, with column headers being Outcome, Odds ratio (95% CI), and P-value. Key findings include significantly higher odds of glucocorticoid use in both men and women with psychiatric disorders. The association is significant among individuals under 65 years but not in those 65 years or older. Notably, individuals with underlying chronic pain and those without underlying COPD or asthma also show higher odds of glucocorticoid use.

Navigate back to Table 4.
