# Supplementary material for: Disparities in oral glucocorticoid prescribing among patients with mental disorders: nationwide cohort study
Source: BJPsych Open. 2026 Jul 3;12(4):e173. doi: 10.1192/bjo.2026.12027 (PMC13359048; doi:10.1192/bjo.2026.12027)
Supplement: Oh and Song supplementary material 3 — Oh and Song supplementary material [file S2056472426120274sup003.docx]

Table S3. The ICD-10 codes of underlying muscloskeletal diseases, autoimmine diseases, and clinical indications and metabolic status

| Diseases | ICD-10 codes |
| --- | --- |
| Fibromyalgia | M79.7 |
| Chronic low back pain | M54.5 (Low back pain), G89.2 (Chronic pain, not elsewhere classified) |
| Other chronic spine pain | M54.2 (Cervicalgia), M54.6 (Pain in thoracic spine), M54.8 (Other dorsalgia), M54.9 (Dorsalgia, unspecified) |
| Degenerative OA | M15.* (Polyarthrosis), M16.* (Hip OA), M17.* (Knee OA), M18.* (First carpometacarpal OA), M19.* (Other/unspecified OA) |
| RA | M05.* (Seropositive RA), M06.* (Other RA) |
| Headache | G43.* (Migraine), G44.* (Other headache syndromes), R51 (Headache, unspecified) |
| Trigeminalgia | G50.0 |
| Myofascial pain | M79.1 (Myalgia, myofascial pain syndrome) |
| Ankylosing spondylitis | M45 |
| Autoimmune hepatitis | K75.4 |
| Behçet’s disease | M35.2 |
| Crohn’s disease | K50.x |
| Polymyositis | M33.2 |
| Polyarteritis nodosa and related conditions | M30.x |
| Sjögren syndrome | M35.0 |
| Systemic lupus erythematosus | M32.x |
| Systemic sclerosis | M34.x |
| Ulcerative colitis | K51.x |
| Asthma | J45.x, J46.x |
| COPD | J44.x |
| Obesity | E66.x |
| Dyslipidemia | E78.x |
| Other metabolic disorders | E88.x |

ICD-10, International Statistical Classification of Diseases and Related Health Problems 10th Revision; RA, rheumatoid arthritis; OA, osteoarthritis; COPD, chronic obstructive pulmonary disease
