# Supplementary material for: Disparities in oral glucocorticoid prescribing among patients with mental disorders: nationwide cohort study
Source: BJPsych Open. 2026 Jul 3;12(4):e173. doi: 10.1192/bjo.2026.12027 (PMC13359048; doi:10.1192/bjo.2026.12027)
Supplement: Oh and Song supplementary material 4 — Oh and Song supplementary material [file S2056472426120274sup004.docx]

Table S4. Baseline demographic and clinical features of participants following propensity score matching including healthcare utilization frequency

| Variable | | | Before PS matching | | ASD | After PS matching | | ASD |  |
| --- | --- | --- | --- | --- | --- | --- | --- | --- | --- |
|  |  |  | PY group  n= 331,020 | Non-PY group  n= 668,980 |  | PY group  n= 249,437 | Non-PY group  n= 249,437 |  |  |
| Age, year | | | 60.8 (18.9) | 50.6 (17.9) | 0.554 | 59.9 (18.9) | 58.9 (18.0) | 0.050 |  |
| Gender: male | | | 159,105 (48.1) | 340,895 (51.0) | 0.058 | 119,805 (48.0) | 118,811 (47.6) | 0.009 |  |
| Residence | | |  |  | 0.019 |  |  | 0.010 |  |
|  | Urban area | | 139,397 (42.1) | 288,048 (43.1) |  | 104,950 (42.1) | 105,975 (42.5) |  |  |
|  | Rural area | | 191,623 (57.9) | 380,932 (56.9) |  | 144,487 (57.9) | 143,462 (57.5) |  |  |
| Household income level | | |  |  | 0.391 |  |  | 0.099 |  |
|  | Medical aid program | | 41,777 (12.6) | 18,222 ( 2.7) |  | 24,968 (10.0) | 15,734 ( 6.3) |  |  |
|  | Q1 (lowest) | | 64,800 (19.6) | 138,364 (20.7) |  | 49,775 (20.0) | 51,682 (20.7) |  |  |
|  | Q2 | | 52,833 (16.0) | 130,831 (19.6) |  | 41,525 (16.6) | 43,380 (17.4) |  |  |
|  | Q3 | | 63,722 (19.3) | 159,608 (23.9) |  | 50,353 (20.2) | 52,746 (21.1) |  |  |
|  | Q4 (highest) | | 102,907 (31.1) | 208,409 (31.2) |  | 78,871 (31.6) | 81,686 (32.7) |  |  |
|  | Unknown | | 4,981 (1.5) | 13,546 (2.0) |  | 3,945 (1.6) | 4,209 (1.7) |  |  |
| Having a job | | | 188,222 (56.9) | 449,213 (67.1) | 0.213 | 141,930 (56.9) | 149,413 (59.9) | 0.061 |  |
| Underlying disability | | |  |  | 0.391 |  |  | 0.092 |  |
|  | Mild to moderate | | 30,545 (9.2) | 26,137 ( 3.9) |  | 20,898 (8.4) | 18,362 ( 7.4) |  |  |
|  | Severe | | 28,478 (8.6) | 12,257 ( 1.8) |  | 17,212 (6.9) | 10,592 ( 4.2) |  |  |
| CCI, point | | | 3.6 (3.1) | 1.9 (2.4) | 0.621 | 3.4 (3.0) | 3.2 (2.8) | 0.078 |  |
|  | Myocardial infarction | | 15,083 (4.6) | 13,565 (2.0) | 0.142 | 10,530 (4.2) | 9,180 (3.7) | 0.030 |  |
|  | Congestive heart failure | | 59,254 (17.9) | 48,574 (7.3) | 0.325 | 40,710 (16.3) | 35,380 (14.2) | 0.057 |  |
|  | Peripheral vascular disease | | 94,724 (28.6) | 93,033 (13.9) | 0.365 | 67,069 (26.9) | 62,096 (24.9) | 0.046 |  |
|  | Cerebrovascular disease | | 84,174 (25.4) | 60,944 ( 9.1) | 0.442 | 57,491 (23.0) | 48,516 (19.5) | 0.088 |  |
|  | Dementia | | 70,291 (21.2) | 33,363 ( 5.0) | 0.496 | 43,610 (17.5) | 31,305 (12.6) | 0.099 |  |
|  | Chronic pulmonary disease | | 138,721 (41.9) | 187,141 (28.0) | 0.294 | 100,692 (40.4) | 96,196 (38.6) | 0.037 |  |
|  | Rheumatic disease | | 36,529 (11.0) | 41,716 ( 6.2) | 0.171 | 26,153 (10.5) | 24,915 (10.0) | 0.016 |  |
|  | Peptic ulcer disease | | 120,023 (36.3) | 154,503 (23.1) | 0.291 | 87,711 (35.2) | 84,745 (34.0) | 0.025 |  |
|  | Mild liver disease | | 155,923 (47.1) | 197,606 (29.5) | 0.367 | 113,552 (45.5) | 110,834 (44.4) | 0.022 |  |
|  | DM without chronic complication | | 144,752 (43.7) | 162,503 (24.3) | 0.419 | 104,231 (41.8) | 99,364 (39.8) | 0.040 |  |
|  | DM with chronic complication | | 42,237 (12.8) | 38,166 ( 5.7) | 0.246 | 29,514 (11.8) | 26,552 (10.6) | 0.038 |  |
|  | Hemiplegia or paraplegia | | 10,247 (3.1) | 6,403 ( 1.0) | 0.152 | 7,047 (2.8) | 5,343 (2.1) | 0.044 |  |
|  | Renal disease | | 18,725 (5.7) | 16,327 ( 2.4) | 0.164 | 12,933 (5.2) | 11,252 (4.5) | 0.031 |  |
|  | Cancer | | 50,431 (15.2) | 59,928 (9.0) | 0.193 | 36,542 (14.6) | 35,405 (14.2) | 0.013 |  |
|  | Moderate or severe liver disease | | 2,445 (0.7) | 2,136 (0.3) | 0.058 | 1,745 (0.7) | 1,527 (0.6) | 0.011 |  |
|  | Metastatic solid tumor | | 6,027 (1.8) | 7,556 (1.1) | 0.057 | 4,509 (1.8) | 4,745 (1.9) | 0.007 |  |
|  | HIV/AIDS | | 511 (0.2) | 567 (0.1) | 0.020 | 404 (0.2) | 372 (0.1) | 0.003 |  |
| Underlying MSD | | |  |  |  |  |  |  |  |
|  | Fibromyalgia | | 11,173 (3.4) | 9,878 (1.5) | 0.124 | 7,610 (3.1) | 6,718 (2.7) | 0.021 |  |
|  | Chronic low back pain | | 108,010 (32.6) | 148,453 (22.2) | 0.236 | 78,817 (31.6) | 76,940 (30.8) | 0.016 |  |
|  | Other chronic spine pain | | 62,020 (18.7) | 84,012 (12.6) | 0.171 | 45,314 (18.2) | 44,674 (17.9) | 0.007 |  |
|  | Degenerative OA | | 123,243 (37.2) | 148,861 (22.3) | 0.332 | 89,079 (35.7) | 85,818 (34.4) | 0.027 |  |
|  | Rheumatoid arthritis | | 21,730 (6.6) | 26,253 (3.9) | 0.119 | 15,586 (6.2) | 15,257 (6.1) | 0.005 |  |
|  | Headache | | 53,819 (16.3) | 54,081 ( 8.1) | 0.252 | 37,993 (15.2) | 34,992 (14.0) | 0.034 |  |
|  | Trigeminalgia | | 4,834 (1.5) | 4,287 (0.6) | 0.080 | 3,341 (1.3) | 2,928 (1.2) | 0.015 |  |
|  | Myofascial pain | | 118,029 (35.7) | 187,197 (28.0) | 0.165 | 87,391 (35.0) | 87,254 (35.0) | 0.001 |  |
| Other analgesics use | | |  |  |  |  |  |  |  |
|  | Paracetamol | | 115,615 (34.9) | 146,686 (21.9) | 0.291 | 83,548 (33.5) | 80,659 (32.3) | 0.025 |  |
|  | NSAIDs | | 24,745 (7.5) | 35,121 (5.2) | 0.091 | 18,064 (7.2) | 17,630 (7.1) | 0.007 |  |
| Other Potential GC indication | | |  |  |  |  |  |  |  |
|  | Ankylosing spondylitis | | 3,185 (1.0) | 3,988 ( 0.6) | 0.042 | 2,305 (0.9) | 2,301 (0.9) | <0.001 |  |
|  | Autoimmune hepatitis | | 1,985 (0.6) | 2,415 (0.4) | 0.035 | 1,428 (0.6) | 1,553 (0.6) | 0.007 |  |
|  | Bechet’s disease | | 602 (0.2) | 624 (0.1) | 0.024 | 460 (0.2) | 390 (0.2) | 0.007 |  |
|  | Crohn’s disease | | 718 (0.2) | 958 (0.1) | 0.017 | 511 (0.2) | 473 (0.2) | 0.003 |  |
|  | Polymyositis | | 394 (0.1) | 395 (0.1) | 0.020 | 263 (0.1) | 265 (0.1) | <0.001 |  |
|  | Polyarteritis | | 427 (0.1) | 455 (0.1) | 0.019 | 308 (0.1) | 316 (0.1) | 0.001 |  |
|  | Shogren syndrome | | 2,372 (0.7) | 1,813 (0.3) | 0.064 | 1,616 (0.6) | 1,227 (0.5) | 0.021 |  |
|  | Systemic lupus erythematosus | | 3,133 (0.9) | 3,703 (0.6) | 0.046 | 2,220 (0.9) | 2,378 (1.0) | 0.007 |  |
|  | Systemic sclerosis | | 203 (0.1) | 237 (0.0) | 0.012 | 149 (0.1) | 162 (0.1) | 0.002 |  |
|  | Ulcerative colitis | | 1,288 (0.4) | 1,694 (0.3) | 0.024 | 924 (0.4) | 913 (0.4) | 0.001 |  |
|  | Obesity | | 1040 (0.3) | 961 (0.1) | 0.036 | 717 (0.3) | 665 (0.3) | 0.004 |  |
|  | Dyslipidemia | | 194,319 (58.7) | 222,543 (33.3) | 0.528 | 141,658 (56.8) | 136,935 (54.9) | 0.038 |  |
|  | Other metabolic disorders | | 1,540 (0.5) | 1,214 (0.2) | 0.050 | 1,074 (0.4) | 891 (0.4) | 0.012 |  |
|  | Asthma | | 36,200 (10.9) | 34,355 (5.1) | 0.215 | 25,081 (10.1) | 21,740 (8.7) | 0.046 |  |
|  | COPD | | 11,079 (3.3) | 7,397 (1.1) | 0.152 | 7,461 (3.0) | 5,914 (2.4) | 0.038 |  |
| Outpatients clinic visits | | |  |  | 1.007 |  |  | 0.098 |  |
|  | | Q1 (0-9) | 34,049 (10.3) | 279,110 (41.7) |  | 32,945 (13.2) | 28,036 (11.2) |  |  |
|  | | Q2 (10-19) | 49,493 (15.0) | 169,358 (25.3) |  | 46,151 (18.5) | 50,972 (20.4) |  |  |
|  | | Q3 (20-44) | 87,785 (26.5) | 144,262 (21.6) |  | 76,673 (30.7) | 94,203 (37.8) |  |  |
|  | | Q4 (≥ 45) | 159,693 (48.2) | 76,250 (11.4) |  | 93,668 (37.6) | 76,226 (30.6) |  |  |

PY, psychiatric disorder; PS, propensity score; ASD, absolute standardized mean difference; CCI, Charlson comorbidity index; DM, diabetes mellitus; AIDS, acquired immunodeficiency syndrome; HIV, human immunodeficiency virus; MSD, musculoskeletal disease; OA, osteoarthritis; NSAIDs, nonsteroidal anti-inflammatory drugs; COPD, chronic obstructive pulmonary disease
