# Supplementary material for: Disparities in oral glucocorticoid prescribing among patients with mental disorders: nationwide cohort study
Source: BJPsych Open. 2026 Jul 3;12(4):e173. doi: 10.1192/bjo.2026.12027 (PMC13359048; doi:10.1192/bjo.2026.12027)
Supplement: Oh and Song supplementary material 5 — Oh and Song supplementary material [file S2056472426120274sup005.docx]

Table S5. Sensitivity analysis of the association between psychiatric disorders and glucocorticoid use using propensity score matching adjusted for healthcare utilization

| Outcome | | Event (%) | OR (95% CI) | *P*-value |
| --- | --- | --- | --- | --- |
| Before PS matching | |  |  |  |
| GC use | |  |  |  |
|  | Non-PY group | 72,130/668,980 (10.8) | 1 |  |
|  | PY group | 45,664/331,020 (13.8) | 1.32 (1.31, 1.34) | <0.001 |
| After PS matching | |  |  |  |
| GC use | |  |  |  |
|  | Non-PY group | 33,411/249,437 (13.4) | 1 |  |
|  | PY group | 37,017/249,437 (14.8) | 1.13 (1.11, 1.15) | <0.001 |

PS, propensity score; OR, odds ratio; CI, confidence interval; PY, psychiatric disorder; GC, glucocorticoid
