# Supplementary material for: Disparities in oral glucocorticoid prescribing among patients with mental disorders: nationwide cohort study
Source: BJPsych Open. 2026 Jul 3;12(4):e173. doi: 10.1192/bjo.2026.12027 (PMC13359048; doi:10.1192/bjo.2026.12027)
Supplement: Oh and Song supplementary material 6 — Oh and Song supplementary material [file S2056472426120274sup006.docx]

Table S6. All ORs with 95% CIs of other covariates in model 1

| Variable | | OR (95% CI) | *P*-value |
| --- | --- | --- | --- |
| Age, year | | 0.99 (0.99, 0.99) | <0.001 |
| Gender: male | | 1.13 (1.11, 1.14) | <0.001 |
| Residence | |  |  |
|  | Urban area | 1 |  |
|  | Rural area | 1.10 (1.09, 1.12) | <0.001 |
| Household income level | |  |  |
|  | Medical aid program | 0.89 (0.87, 0.92) | <0.001 |
|  | Q1 (lowest) | 1 |  |
|  | Q2 | 1.02 (1.00, 1.04) | 0.123 |
|  | Q3 | 1.01 (0.98, 1.02) | 0.735 |
|  | Q4 (highest) | 1.01 (0.99, 1.02) | 0.611 |
|  | Unknown | 0.97 (0.92, 1.02) | 0.224 |
| Having a job | | 1.06 (1.04, 1.07) | <0.001 |
| Underlying disability | |  |  |
|  | Mild to moderate | 1.00 (0.97, 1.02) | 0.996 |
|  | Severe | 0.81 (0.78, 0.84) | <0.001 |
| CCI, point | | 1.02 (1.02, 1.02) | <0.001 |
| Underlying MSD | |  |  |
|  | Fibromyalgia | 1.14 (1.10, 1.18) | <0.001 |
|  | Chronic low back pain | 1.15 (1.13, 1.17) | <0.001 |
|  | Other chronic spine pain | 1.15 (1.13, 1.17) | <0.001 |
|  | Degenerative OA | 1.33 (1.31, 1.35) | <0.001 |
|  | Rheumatoid arthritis | 1.77 (1.73, 1.81) | <0.001 |
|  | Headache | 1.11 (1.09, 1.13) | <0.001 |
|  | Trigeminalgia | 1.21 (1.15, 1.27) | <0.001 |
|  | Myofascial pain | 1.31 (1.29, 1.33) | <0.001 |
| Other analgesics use | |  |  |
|  | Paracetamol | 1.96 (1.93, 1.99) | <0.001 |
|  | NSAIDs | 1.82 (1.79, 1.86) | <0.001 |
| Underlying autoimmune disease | |  |  |
|  | Ankylosing spondylitis | 1.46 (1.38, 1.55) | <0.001 |
|  | Autoimmune hepatitis | 1.30 (1.20, 1.41) | <0.001 |
|  | Bechet’s disease | 3.59 (3.18, 4.05) | <0.001 |
|  | Crohn’s disease | 1.33 (1.17, 1.51) | <0.001 |
|  | Polymyositis | 1.21 (1.02, 1.44) | 0.029 |
|  | Polyarteritis | 1.75 (1.50, 2.05) | <0.001 |
|  | Shogren syndrome | 1.43 (1.33, 1.55) | <0.001 |
|  | Systemic lupus erythematosus | 1.66 (1.57, 1.76) | <0.001 |
|  | Systemic sclerosis | 1.35 (1.09, 1.68) | 0.007 |
|  | Ulcerative colitis | 1.50 (1.36, 1.65) | <0.001 |
|  | Obesity | 1.30 (1.05, 1.61) | 0.018 |
|  | Dyslipidemia | 1.21 (1.02, 1.43) | 0.027 |
|  | Other metabolic disorders | 1.26 (1.11, 1.42) | <0.001 |
|  | Asthma | 1.42 (1.34, 1.50) | <0.001 |
|  | COPD | 1.73 (1.48, 2.02) | <0.001 |

OR, odds ratio; CI, confidence interval; CCI, Charlson comorbidity index; DM, diabetes mellitus; AIDS, acquired immunodeficiency syndrome; HIV, human immunodeficiency virus; MSD, musculoskeletal disease; OA, osteoarthritis; NSAIDs, nonsteroidal anti-inflammatory drugs; COPD, chronic obstructive pulmonary disease
